# Three-Dimensional Light Sheet Fluorescence Microscopy of Lungs To Dissect Local Host Immune-Aspergillus fumigatus Interactions

**DOI:** 10.1128/mBio.02752-19

**Published:** 2020-02-04

**Authors:** Jorge Amich, Zeinab Mokhtari, Marlene Strobel, Elena Vialetto, Dalia Sheta, Yidong Yu, Julia Hartweg, Natarajaswamy Kalleda, Katja J. Jarick, Christian Brede, Ana-Laura Jordán-Garrote, Sina Thusek, Katharina Schmiedgen, Berkan Arslan, Jürgen Pinnecker, Christopher R. Thornton, Matthias Gunzer, Sven Krappmann, Hermann Einsele, Katrin G. Heinze, Andreas Beilhack

**Affiliations:** a Department of Medicine II and IZKF Research Laboratory, Würzburg University Hospital, Würzburg, Germany; b Research Center for Infectious Diseases, Julius-Maximilians-University Würzburg, Würzburg, Germany; c Rudolf Virchow Center, Julius-Maximilians-University Würzburg, Würzburg, Germany; d Institute for Experimental Immunology and Imaging, University Hospital, University Duisburg-Essen, Essen, Germany; e Biosciences, University of Exeter, Exeter, United Kingdom; f Institute of Clinical Microbiology, Immunology and Hygiene, Universitätsklinikum Erlangen and Friedrich-Alexander-Universität (FAU) Erlangen-Nürnberg, Erlangen, Germany; Universidade de Sao Paulo

**Keywords:** *Aspergillus fumigatus*, invasive aspergillosis, host-pathogen interactions, lung infection, lung immunity, microscopy/imaging, host immune response, light sheet fluorescence microscopy, murine models of invasive pulmonary aspergillosis, *in vivo* fungal growth, whole-organ imaging

## Abstract

Aspergillus fumigatus is an opportunistic fungal pathogen that can cause life-threatening invasive lung infections in immunodeficient patients. The cellular and molecular processes of infection during onset, establishment, and progression of A. fumigatus infections are highly complex and depend on both fungal attributes and the immune status of the host. Therefore, preclinical animal models are of paramount importance to investigate and gain better insight into the infection process. Yet, despite their extensive use, commonly employed murine models of invasive pulmonary aspergillosis are not well understood due to analytical limitations. Here, we present quantitative light sheet fluorescence microscopy (LSFM) to describe fungal growth and the local immune response in whole lungs at cellular resolution within its anatomical context. We analyzed three very common murine models of pulmonary aspergillosis based on immunosuppression with corticosteroids, chemotherapy-induced leukopenia, or myeloablative irradiation. LSFM uncovered distinct architectures of fungal growth and degrees of tissue invasion in each model. Furthermore, LSFM revealed the spatial distribution, interaction, and activation of two key immune cell populations in antifungal defense: alveolar macrophages and polymorphonuclear neutrophils. Interestingly, the patterns of fungal growth correlated with the detected effects of the immunosuppressive regimens on the local immune cell populations. Moreover, LSFM demonstrates that the commonly used intranasal route of spore administration did not result in complete intra-alveolar deposition, as about 80% of fungal growth occurred outside the alveolar space. Hence, characterization by LSFM is more rigorous than by previously used methods employing murine models of invasive pulmonary aspergillosis and pinpoints their strengths and limitations.

## Abstract (precis)

The use of animal models of infection is essential to advance our understanding of the complex host-pathogen interactions that take place during Aspergillus fumigatus lung infections. As in the case of humans, mice need to suffer an immune imbalance in order to become susceptible to invasive pulmonary aspergillosis (IPA), the most serious infection caused by A. fumigatus. There are several immunosuppressive regimens that are routinely used to investigate fungal growth and/or immune responses in murine models of invasive pulmonary aspergillosis. However, the precise consequences of the use of each immunosuppressive model for the local immune populations and for fungal growth are not completely understood. Here, to pin down the scenarios involving commonly used IPA models, we employed light sheet fluorescence microscopy (LSFM) to analyze whole lungs at cellular resolution. Our results will be valuable to optimize and refine animal models to maximize their use in future research.

## INTRODUCTION

Aspergillus fumigatus is a filamentous, spore-producing, saprotrophic fungus abundant in the natural environment. Airborne A. fumigatus spores, termed conidia, can easily penetrate the human respiratory tract and reach the lung alveoli due to their small size (2 to 3 μm). This rarely has consequences in immunocompetent individuals as innate immune defense mechanisms can very efficiently eliminate conidia. However, A. fumigatus can colonize and invade human lung tissues of susceptible hosts with an impaired or imbalanced immune system, causing a spectrum of diseases collectively named aspergillosis (1, 2). On a worldwide basis, A. fumigatus is the most prominent fungal pathogen of the human lung and is responsible for an estimated incidence of more than 300,000 cases of invasive aspergillosis, a lethal disease that causes ∼150,000 deaths annually (3).

Fungal attributes that enable A. fumigatus to thrive in the tissues (metabolic versatility, resistance to stress, etc.) as well as the host immune status that triggers susceptibility (immunosuppression, underlying diseases, etc.) influence the infection process. *In vitro* experimentation has greatly contributed to our understanding of many details in immune-A. fumigatus interactions. However, *in vitro* assays cannot achieve a comprehensive picture of the dynamic and complex host pathogen interactions, which justifies studying this multilayered interplay in *in vivo* models of infection. Particularly, mouse infection models have provided important insights into host-fungal *in vivo* interactions and the pathophysiology of the infection process (4). Yet, precise information about the spatiotemporal evolution of the local host-pathogen interactions has been limited in these animal models. Classic and fluorescence microscopy technologies allow visualization of the interaction of host and fungal cells in the infected organ; however, as the tissue needs to be sectioned into micrometer-thin slices, the analysis can be performed only on small areas. As a result, analyses of histologic specimens suffer from a sampling bias, the overall three-dimensional (3D) anatomical context can be lost, the information obtained is fragmented, and the value of data quantification is limited. Noninvasive bioluminescence imaging (BLI) has allowed spatiotemporal tracking of pathogen growth or immune cell recruitment in living animals (5, 6). Yet, to date BLI has not provided the optical resolution needed to investigate interactions on the single-cell level and consequently often requires complementary approaches for more-refined analyses. One technique commonly used is flow cytometry, which permits quantification of host cell populations in a high-throughput manner (7). Despite this, analysis of cell populations with flow cytometry requires organs to be processed into single-cell suspensions. Disruption of solid organs and subsequent cell isolation may affect the accurate representation of certain cell subpopulations, often resulting in deviations with respect to the extracted cell types, which necessitate high numbers of research animals to obtain reliable results. Furthermore, flow cytometry has serious drawbacks for the analysis of filamentous fungi (8) and is completely blind for the anatomical context.

A common feature of all A. fumigatus infections is the capacity of inhaled spores to persist and grow in the human respiratory tract. Therefore, to maximize the value of preclinical *in vivo* animal models for improving our understanding of host-pathogen interactions, it is crucial to study the structural organization of fungal spread and the ensuing immune response in the context of the complex spatial microarchitecture of the lung.

Recent advances in light sheet fluorescence microscopy (LSFM) for deep tissue analyses have enormously extended the tool box for studying biological processes (9, 10). Previously, we employed LSFM to interrogate distributions and interactions in inflammation, cancer, and hematopoiesis in whole organs at cellular resolution (11–14). In the present study, we applied LSFM to investigate the complex spatial relationships underlying A. fumigatus-host interactions in lungs of mice under different immunosuppressive regimens. We quantified and visualized fungal growth in the three-dimensional anatomical context of whole lungs. Concurrently, we defined the immune response in its anatomical context and quantified host-pathogen interactions within the intact three-dimensional (3D) tissue microenvironment of the lung. Following the fundamental tenet that structure and function are inextricably linked, LSFM has proved to be an excellent technique to decipher local host-pathogen interactions in the defined structural context of whole organs at cellular resolution.

## RESULTS

### Light sheet fluorescent microscopy (LSFM) visualization of whole lungs at cellular resolution.

**FIG 1:**
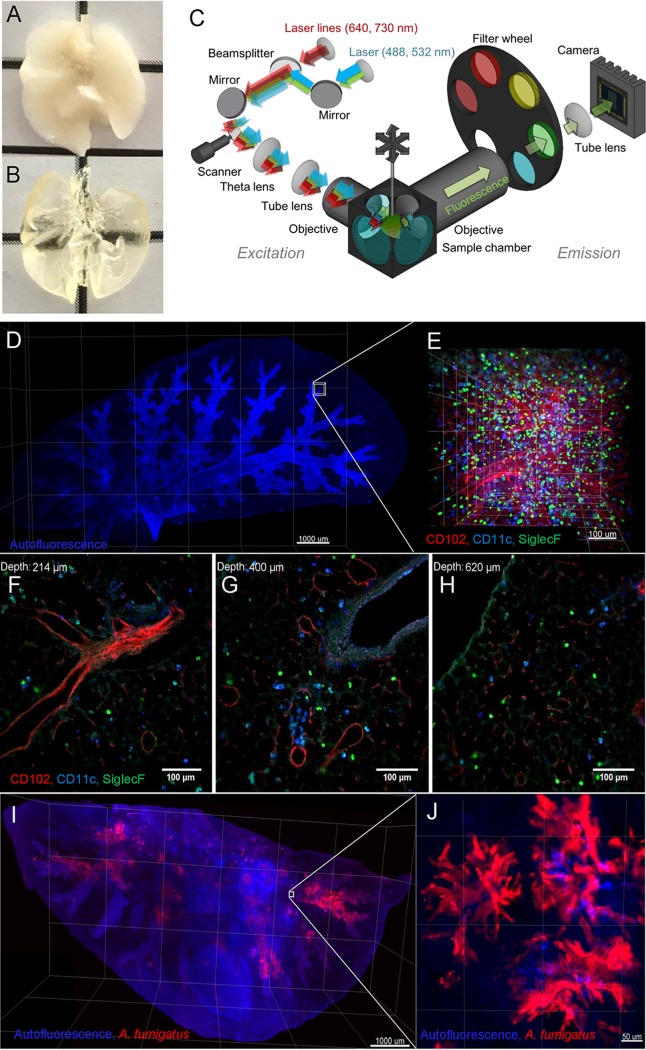
Light sheet fluorescence microscopy (LSFM) maps the three-dimensional structural microarchitecture of intact lungs with cellular resolution. (A and B) Whole lungs explanted from a perfused mouse before (A) and after (B) clearing. (C) Optical setup of LSFM. (D) Autofluorescence signal of a murine lung lobe acquired with LSFM. Several fields of view were acquired and stitched together (5× objective, scale bar = 1 mm). (E) Zoomed LSFM image precisely depicts immune cell subpopulations at cellular resolution (20× objective, scale bar = 100 μm). Alveolar macrophages (SiglecF^+^ CD11c^+^, light blue) are evenly distributed in the anatomical microenvironment of the lung. (SiglecF^−^ CD11c^+^ cells correspond predominantly to dendritic cells and SiglecF^+^ CD11c^−^ cells to eosinophils.) CD102^+^ blood vessels are depicted in red. (F, G, and H) Representative two-dimensional optical sections of the lung of an imaging stack at penetration depths of 214 μm (F), 400 μm (G), and 620 μm (H). (I) Lung lobe of a neutropenic mouse infected with 2 × 10^5^ A. fumigatus conidia (5× objective, scale bar = 1 mm). (J) Foci of fungal growth (red) can be observed throughout the tissue as intense cloudy signals at the terminal bronchi (not detected with isotype control antibody) (20× objective, scale bar = 50 μm).

To enable deep-tissue microscopy, we have successfully adapted our previously published clearing protocol (11) for use in whole lungs (Fig. 1A and B). Employing a custom-built LSFM setup (Fig. 1C), we imaged entire lung lobes with cellular resolution. Intrinsic autofluorescence, detected in the range of 500 to 550 nm, provided anatomical details of the lung airways, revealing the structure of bronchi and bronchioles (Fig. 1D, blue). To accurately detect specific immune cell populations, we employed combinations of fluorescently labeled antibodies. For instance, to visualize CD11c^+^ SiglecF^+^ alveolar macrophages (AMs) (Fig. 1E), we detected the combined signals from cells stained with anti-CD11c antibodies conjugated to Alexa Fluor 532 (A532) and anti-SiglecF antibodies conjugated to DyLight 755. Additionally, we imaged blood vessels with an anti-CD102 antibody conjugated to Alexa Fluor 488 (A488) to visualize AMs and neutrophil granulocytes (polymorphonuclear leukocytes [PMNs]; CD11b^+^ Ly6G^+^) in the context of the pulmonary vasculature. Optical scanning with LSFM enabled us to interrogate different depths of the lung tissue (Fig. 1F to H; see also Movie S1 in the supplemental material) and subsequently to render series of two-dimensional images into a 3D movie (Movie S2). To detect fungal growth within the lung, we utilized the *Aspergillus*-specific monoclonal antibody JF5 conjugated with DyLight 650 dye (Fig. 1I and J), which specifically targets an *Aspergillus* mannoprotein antigen secreted during active growth (15). A. fumigatus infection caused disruption of the lung tissue and the appearance of dead cells, which manifested as an increase of tissue autofluorescence and blurring of the observed granular details of well-defined epithelial barriers in healthy mice.

10.1128/mBio.02752-19.5 MOVIE S1 Optical sectioning of an intact lung lobe with multicolor LSFM. Download Movie S1, AVI file, 5.6 MB. Copyright © 2020 Amich et al. 2020 Amich et al. This content is distributed under the terms of the Creative Commons Attribution 4.0 International license.

10.1128/mBio.02752-19.6 MOVIE S2 Virtual journey through an intact lung lobe with multicolor LSFM of healthy and A. fumigatus-infected mice. Download Movie S2, AVI file, 18.0 MB. Copyright © 2020 Amich et al. 2020 Amich et al. This content is distributed under the terms of the Creative Commons Attribution 4.0 International license.

### LSFM reveals the architecture of fungal growth under different immunosuppressive regimens.

**FIG 2:**
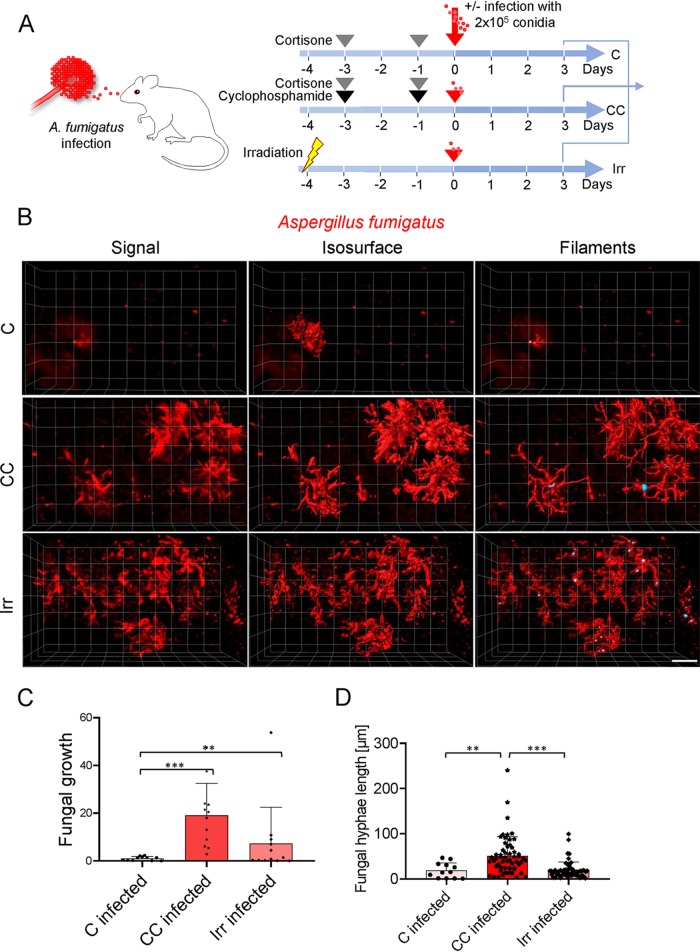
The level of immunosuppression determines local fungal spread. (A) Mouse models of immunosuppression employed to study invasive aspergillosis. (B) LSFM reveals 3D structures of fungal growth in different models of immunosuppression. Images represent fluorescence signal detected with LSFM (left), calculated isosurfaces of fungal hyphae formation (middle), and filament allocation (right) for a representative focus of fungal growth in each immunosuppression model (20× objective, scale bar = 100 μm) (C) Percentage of fungal mass infiltrating lung tissue. Lungs were imaged at 5 to 6 locations to cover the whole lung lobule, and two mice per condition were analyzed. (D) Length of all A. fumigatus filaments (hyphae) detected in the lungs of two mice per condition were analyzed. For data presented in all graphs, One-way ANOVA with multiple comparisons was applied. (***, *P* < 0.0001; **, 0.01* < P* < 0.001).

Murine models of invasive pulmonary aspergillosis (IPA) represent the gold standard to investigate the pathogenic potential of different A. fumigatus isolates or mutant strains *in vivo* and to evaluate the efficacy of antifungal or immunomodulatory treatments (4). In mice, as in humans, immunosuppression increases the risk of development of IPA. To model disease, various immunosuppressive regimens are established, and their use is strongly based on the particular research issue under investigation (for a review, see reference 16). The two most prominent immunosuppression models render the mice either leukopenic (usually with alkylating agents), with the objective of mimicking the responses of profoundly neutropenic patients (e.g., those suffering from leukemia or undergoing hematopoietic stem cell transplantation), or immunomodulated (usually by the injection of steroids), which the aim of mimicking the responses of patients treated with immunomodulatory drugs (e.g., those subjected to solid-organ transplantation or suffering from graft-versus-host disease; see reference 4 and references within). Despite basic knowledge of the action of the immunosuppressive treatments, there are still many gaps in our understanding of their effect on fungal growth such as the exact spatiotemporal orchestration of the humoral and cellular interplay recognizing and controlling inhaled fungal spores under physiological and pathological conditions. Consequently, we imaged and quantified the structural organization of foci of fungal growth in this study and quantified and analyzed sustained defects in the innate immune response at a late stage of infection in three commonly used immunosuppressive regimens (Fig. 2A), all within intact lungs of A. fumigatus-infected mice (Fig. 1). Cortisone (C) is known to impair the action of the immune system without depleting AMs or PMNs (17–22). Notably, under these conditions, LSFM revealed only a few constrained foci of fungal growth (Fig. 2B [top] and D) that covered a small area of the lung (Fig. 2C), and the fungus could form only a few short hyphal filaments (Fig. 2B [top] and D). In the most commonly used leukopenic model (treatment with cyclophosphamide and cortisone [CC]), mice are treated with the alkylating agent cyclophosphamide, which depletes proliferative immune cells, and with two doses of cortisone acetate, impairing the function of resident cells postmitosis (23–31). Under these conditions, *A. fumigatus* invaded the tissue extensively and formed many dense foci of fungal growth (Fig. 2B [middle]) that invaded a greater ratio of lung tissue (Fig. 2C) and produced many long hyphae (Fig. 2B [middle] and D). Myeloablative irradiation (Irr) is used as a conditioning treatment for hematopoietic cell transplantation models (6, 32). This myeloablative conditioning regimen eliminates the host hematopoietic compartment, and yet radiation-resistant tissue-resident immune cells, such as yolk sac-derived alveolar macrophages, can persist for extended time periods (65). After myeloablative irradiation was performed, we observed that A. fumigatus grew extensively (Fig. 2B [bottom]) but that the volume of invaded tissue was 38% lower than that seen with CC treatment (Fig. 2C) and, even in cases in which many filaments were formed, that they were on average 2.6 times shorter than those seen with CC treatment (Fig. 2D). Additionally, the structures of the growing masses differed as they formed more-dispersed mycelia.

We also tested how LSFM compares with the gold standard method for quantification of fungal burden, SYBR green-based quantitative PCR (qPCR). We infected CC-treated mice with A. fumigatus conidia and euthanized them 16 or 72 h after infection. The four lobes of the right lung from each mouse were homogenized, DNA was extracted, the fungal burden was quantified by qPCR (as described in Materials and Methods), and the left lung lobes were analyzed by LSFM. As can be seen in Fig. S1A and B in the supplemental material, both techniques were sensitive enough to detect the fungus 16 h after infection, and the results clearly reflected the enormous increment in fungal burden at day +3; thus, the two techniques were able to reflect the tendency of burdens similarly. While the qPCR results were at the limit of detection at 16 h, LSFM could detect abundant fungal growth (Fig. S1C), suggesting that LSFM is more sensitive than qPCR. Actually, the fact that fungal growth can be imaged represents an additional advantage of LSFM, as the detection of fungal burden at low levels of infection can be visually validated. Importantly, the fungal burdens detected in this independent experiment were comparable to the values shown in Fig. 2C, which corroborates that as LSFM analyzes the entire lung, the data collected appear highly accurate and reproducible. Thus, LSFM helps to reduce the number of replicates required to obtain significant and reliable results.

10.1128/mBio.02752-19.1 FIG S1 Direct comparison of LSFM with qPCR to quantify fungal burden in infected lungs demonstrates the advantages of quantitative microscopy. CC-treated immunosuppressed mice were infected with A. fumigatus and culled 16 or 72 h after infection. (A and B) Fungal burdens of 3 mice per group were analyzed with qPCR (A) or LSFM (2 to 3 3D stacks per mouse) (B), showing similar results with the two techniques. Standard deviations (SD) were higher in qPCR than in LSFM. (C) Whilst qPCR results were close to the limit of detection at 16 h after infection, LSFM could still detect abundant fungal growth. This confirmed the high sensitivity of LSFM in reliably measuring fungal growth in lungs even at early stages of A. fumigatus infection. Data were analyzed using Student *t* tests with Welch’s correction (**, *P < *0.01). Download FIG S1, TIF file, 1.4 MB. Copyright © 2020 Amich et al. 2020 Amich et al. This content is distributed under the terms of the Creative Commons Attribution 4.0 International license.

To this point, LSFM revealed clear differences in fungal growth in the lungs of mice treated with commonly used immunosuppressive regimens and allowed us to map the initiation of IPA in the context of the intact 3D lung environment with high resolution.

### LSFM pinpoints differences in the immune responses between immunosuppressive regimens.

**FIG 3:**
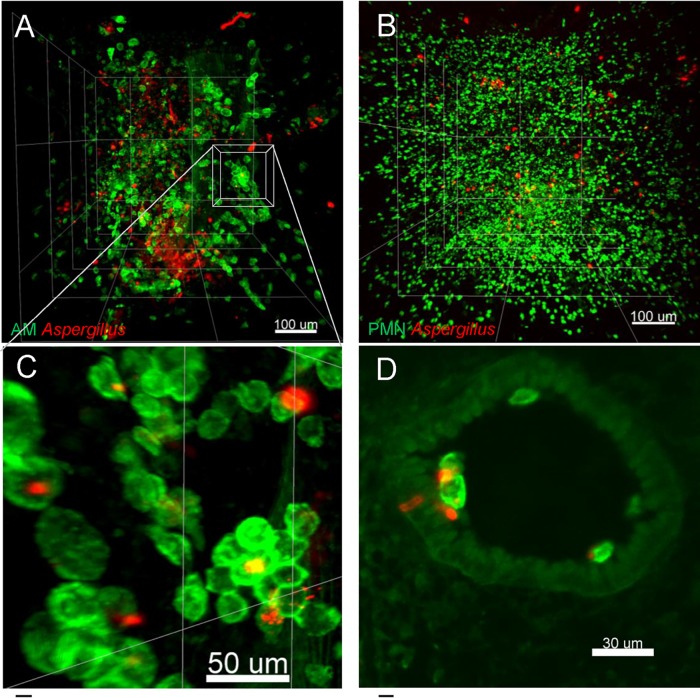

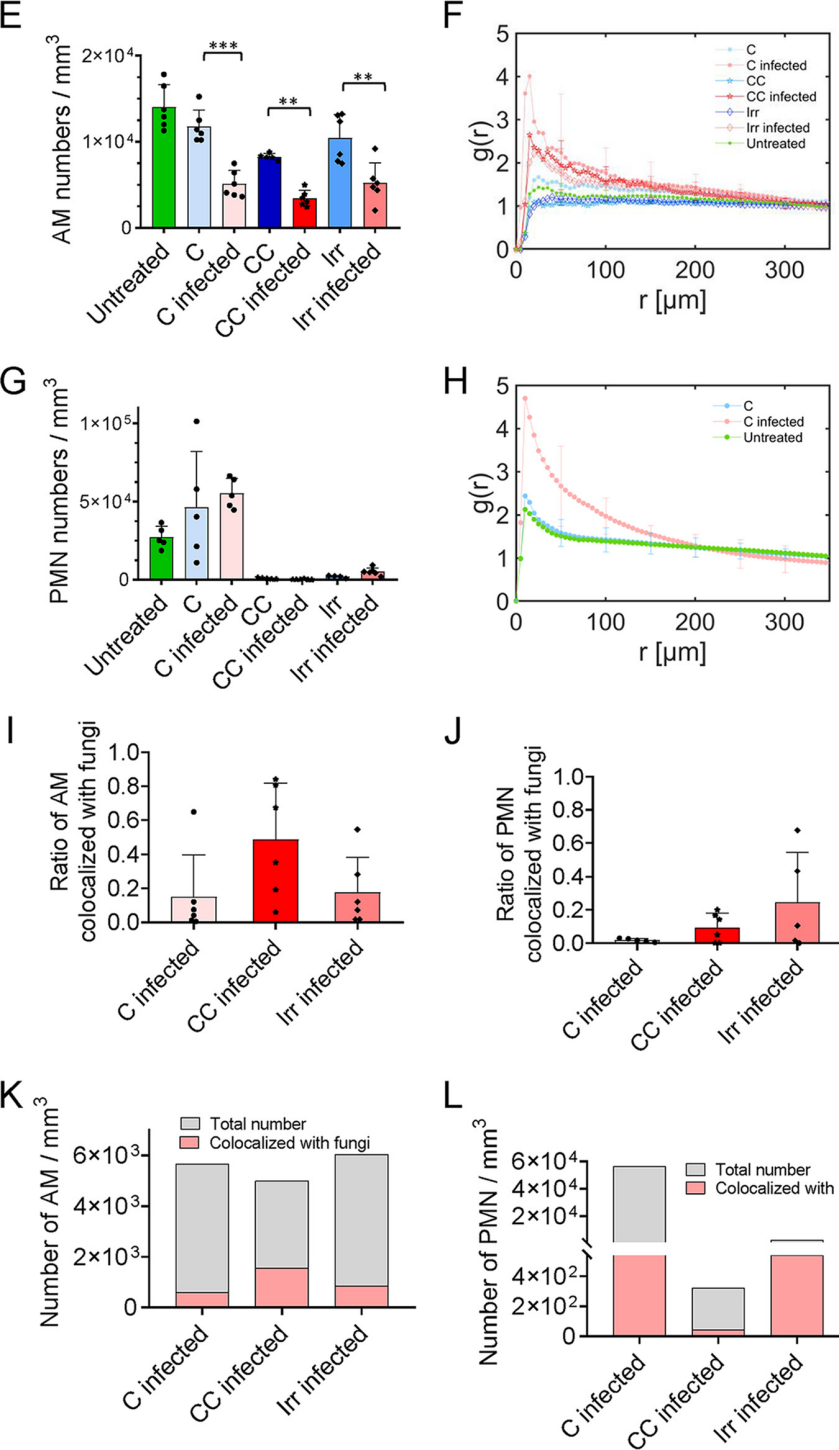
Quantitative image analysis reveals distribution and interaction of immune cells with A. fumigatus in 3D lung environment. (A and B) Representative 3D images acquired and reconstructed from lungs of infected mice treated with cortisone (C) or with cyclophosphamide and cortisone (CC) or after myeloablative whole-body irradiation (Irr). (A) AMs (SiglecF^+^ CD11c^+^; depicted in green) and A. fumigatus (JF5 antibody staining; depicted in red) after irradiation and infection. (B) PMNs (CD11b^+^ Ly6G^+^) in infected mouse treated with cortisone are visualized in green. (C) Magnification of the image in panel A reveals strong AM clustering. (D) Fungal spores that had been engulfed by AMs germinated in close proximity to lung epithelial cells upon infection and irradiation. (E) AM numbers quantified with LSFM image analysis in the lungs of mice before and after A. fumigatus infection under different immunosuppressive regimens. AM numbers did not decrease after C and Irr treatments and declined only slightly after CC treatment. AM numbers markedly declined upon infection. (F) Pair correlation function [*g*(*r*)] calculated for AMs using multiple LSFM images from two mice. Correlation data reveal that the AMs clustered 3 days after infection, suggesting recruitment to sites of A. fumigatus infection (paired signed rank test, *P* < 0.001) (G) Number of PMNs in the lungs of immunosuppressed mice assessed by quantitative analysis of LSFM images. The number of PMNs increased after C treatment and was maintained upon infection. PMNs were virtually eliminated after CC and Irr treatments. (H) Pair correlation function for PMNs was calculated only from mice treated with cortisone only, due to the extremely low numbers in CC and Irr mice (paired signed rank test, *P* < 0.001). PMNs show a clustered distribution upon infection, indicating active recruitment to the infection sites. (I to L) Direct colocalization of AMs and PMNs with A. fumigatus indicates that these immune cells ingested fungal material, supporting the hypothesis of an active immune response against invasive aspergillosis. All quantifications were made from two mice per condition and 2 to 3 3D stacks per mouse. One-way ANOVA with Tukey’s multiple comparisons was applied (**, *P < *0.01; ***, *P* < 0.001).

As LSFM revealed the anatomical information of foci of fungal growth in an unbiased fashion, we explored the specific cellular immune response of AMs and PMNs (Fig. 3A to D) to infection at these hot spots and the impact of common immunosuppressive regimens (Fig. 2A).

Corticosteroid treatment did not affect the quantity of AMs in the lungs, but their number significantly decreased upon infection (Fig. 3E), which suggests that many AMs did not survive the challenge with the fungus. Nevertheless, AMs formed clusters upon infection (Fig. 3F), indicating that the corticosteroid treatment did not impair lung-residing AM recognition of and interaction with A. fumigatus. Corticosteroid treatment significantly increased PMN numbers in the lungs of mice. However, upon infection, total PMN numbers did not show a further increase 3 days after A. fumigatus exposure (Fig. 3G), which suggested that either further PMN recruitment had been impaired or that PMNs died at the same rate as that at which they were being recruited or relocated to other tissue sites (33). Notably, LSFM revealed that PMNs strongly clustered within lung tissue upon infection (Fig. 3H), indicating that corticosteroids did not impair the local recruitment and interaction of these cells with the fungus. We then calculated the number of AMs (Fig. 3I and K) and PMNs (Fig. 3J and L) that directly colocalized with the fungus (IMARIS detection of spatial overlap of three signals, namely, CD11c-SiglecF-JF5 or CD11b-Ly6G-JF5) as a measure of phagocytic activity. We observed only 10% of AMs (53 of 533 AM; Fig. 3K) and <2% of PMNs (102 of 6400 PMNs; Fig. 3L) completely engulfing A. fumigatus, suggesting that under conditions of corticosteroid treatment, these immune cells, although present at the site of infection, were inefficient in eliminating the fungus.

In the leukopenic model, CC treatment slightly decreased AM numbers. A. fumigatus infection further reduced AM numbers, suggesting again that AMs died during A. fumigatus challenge (Fig. 3E). Similarly, as in the corticosteroid model, CC treatment led to AMs gathering in clusters upon infection (Fig. 3F), indicating that lung-residing AMs were not impaired in recognizing A. fumigatus. In stark contrast to the corticosteroid regimen, CC treatment virtually eliminated all PMNs in the lungs with no PMN recruitment after infection, indicative of profound neutropenia. Interestingly, the absolute numbers of AMs (Fig. 3I) that colocalized with the fungus were slightly higher than in the C model (162 of 362 AMs; Fig. 3K), which suggests that the AMs killed more A. fumigatus conidia in this model. However, PMNs were not present to eliminate resilient A. fumigatus spores, which likely accounts for the huge degree of fungal growth in this model (Fig. 2B).

In the irradiation model, infection reduced AM numbers less dramatically than the C and CC treatments (Fig. 3E), confirming the radiation resistance of AMs even after myeloablative irradiation and suggesting that AMs may be more relevant to fight A. fumigatus in this model of suppression. Nevertheless, the number of AMs that had phagocytosed conidia was below 10% (89 of 949 AMs) (Fig. 3I to K), with some engulfed conidia germinating within AMs (Fig. 3D), indicating that irradiation partially impaired the killing potential of AMs. In addition, as in the CC model, the PMNs were almost entirely depleted from the lungs (Fig. 3G), although the very few that remained seemed to be more efficiently fighting the fungus, as reflected by the higher proportion of PMNs (38 of 212 PMNs; Fig. 3L) that colocalized with the fungus, which we propose as the potential reason for the observed reduction in fungal growth (Fig. 2B to D).

To corroborate our assumption that low numbers of biological replicates (i.e., mice) are sufficient to obtain reliable results with LSFM, we performed an independent experiment in which we repeated various of the treatments using a higher number of mice (Fig. S2). Comparison with the results presented in Fig. 3 demonstrated that the observed changes in AM numbers (Fig. S2A), AM clustering (Fig. S2B), and colocalization of AMs with fungi (Fig. S2C and D) could be perfectly reproduced.

10.1128/mBio.02752-19.2 FIG S2 Independent experiment confirming microscopic quantification of AMs within lungs. To evaluate whether quantitative analysis of LSFM images provides accurate and reproducible data representing numbers of immune cells and the response at the site of infection, we conducted an independent experiment with additional experimental mouse replicates (*n* = 3 mice/group and 2 to 3 stacks per mouse). The data representing the number (A), clustering pattern (B), colocalization (C), and ratios (D) of AMs were comparable to those reported in the first experiment (Fig. 3). One-way ANOVA was applied with Tukey’s multiple comparisons. Download FIG S2, TIF file, 0.5 MB. Copyright © 2020 Amich et al. 2020 Amich et al. This content is distributed under the terms of the Creative Commons Attribution 4.0 International license.

Next, we tested how LSFM compares with flow cytometry, the gold standard technique, for quantification of immune cells (Fig. S3). We quantified AMs in immunocompetent mice and in C-treated mice with and without infection. In all cases, the numbers of cells detected with flow cytometry were 5 times lower than those detected with LSFM, reflecting significant underrepresentation of this cell-type with flow cytometry (Fig. S3B). This reduced capacity to detect sufficient cell numbers has important consequences for data analysis and interpretation; in C-treated mice, for instance, the number of AMs significantly decreased upon infection (Fig. 3E; see also Fig. S2A and S3B), but flow cytometry could not detect such reduction (Fig. S3B).

10.1128/mBio.02752-19.3 FIG S3 Direct comparison of LSFM with flow cytometry to quantify immune cells in murine lungs. Levels of AMs in lungs of immunocompetent mice and in C-treated mice (infected and not infected) were quantified by flow cytometry or LSFM (*n* = 3 mice). (A) Representative flow cytometric gating strategy to measure AMs in lungs of immunocompetent mice, C-treated uninfected mice, and C-treated infected mice. (B) Under all conditions, the numbers of AMs consistently measured with flow cytometry (*n*= 3 mice/group and 2 to 3 technical replicates) were 5 times lower than the actual numbers detected with LSFM (*n* = 3 mice/group and 2 to 3 3D stacks per mouse). One-way ANOVA was applied with Tukey’s multiple comparisons (***, *P < *0.001). Download FIG S3, TIF file, 0.7 MB. Copyright © 2020 Amich et al. 2020 Amich et al. This content is distributed under the terms of the Creative Commons Attribution 4.0 International license.

Conclusively, LSFM sensitively revealed changes in the total pulmonary lung environment as well as differences in immune cell activity in locally confined foci of A. fumigatus infection at subcellular resolution in the lungs of mice treated with various immunosuppressive regimens.

### The majority of A. fumigatus conidia do not reach the lung alveoli in the intranasal infection model.

**FIG 4:**
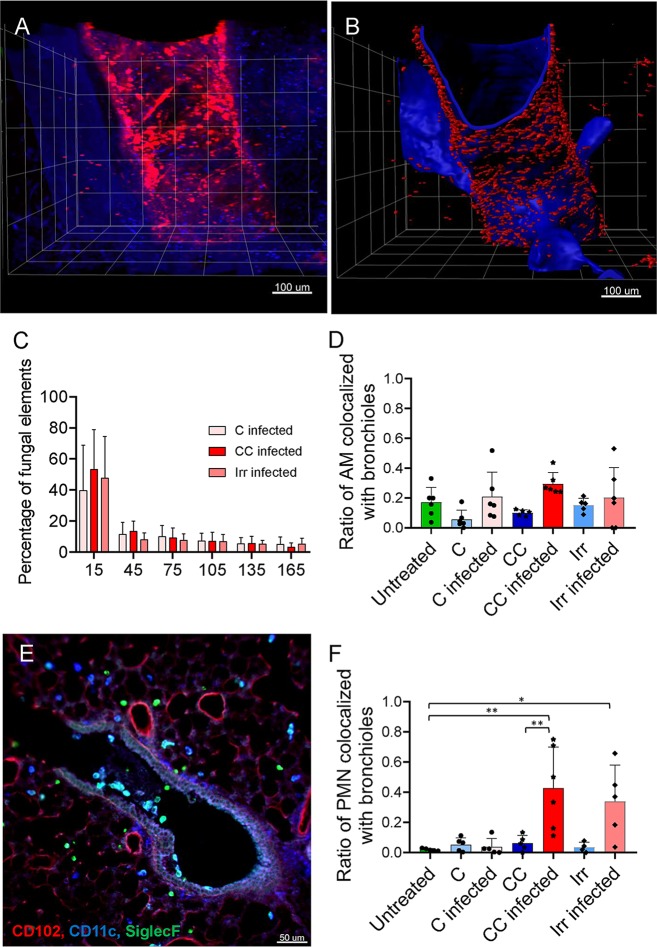
Interaction analysis of fungal burden and bronchioles reveals heterogeneous distribution of fungal spores in murine lungs. (A) 3D representation of A. fumigatus distribution and lung morphology with images acquired and reconstructed from infected lungs. (B) Isosurfaces of fungi and bronchioles were reconstructed from anti-A. fumigatus JF5 antibody signals and antipodoplanin staining. Isosurface data were used to compute distances between fungi and bronchioles of lung. (C) Distance distributions of fungi and bronchioles are presented as a histogram for different treatments. In all treatment groups, a high proportion (∼60%) of spores were found to be in close proximity to bronchioles, indicating that a vast majority of the infective conidia did not reach the alveoli. Quantification (D and F) and imaging (E) representing colocalization of AMs (D and E) and PMNs (F) with bronchioles were performed, and quantification data are shown as ratios of total cell numbers (D and F). Notably, the results revealed a low ratio of AMs localized in the bronchioles under steady-state conditions that increased upon infection for C-treated and CC-treated mice. Furthermore, upon infection, the ratio of PMNs significantly increased in CC-treated and Irr-treated mice. All quantifications were made from two mice per condition and 2 to 3 3D stacks per mouse. One-way ANOVA Tukey’s multiple comparisons was applied (*, *P* < 0.05).

One of the factors considered to be important for A. fumigatus pathogenic potential is the small size of its conidia (2 to 3 μm), which permits their penetration through the human respiratory tract to reach the lung alveoli (31, 32). Therefore, many studies have focused on the interaction of A. fumigatus with alveolar resident cells such as alveolar epithelial cells and alveolar macrophages. However, despite this assumption, from a clinical perspective, IPA was defined previously as the invasion of the pulmonary parenchyma by the growing hyphae of *Aspergillus* (33) and IPA is no longer considered a disease of the alveoli; however, very detailed localization studies have not been done and would be difficult to do in human patients. Therefore, we employed LSFM to reveal spatial information with respect to the distribution of A. fumigatus in the context of the pulmonary microanatomy to address the issue of whether or not the localization of the infective conidia is within the alveoli. To this end, we stained lung epithelial cells with an antipodoplanin antibody, visualizing the epithelial surface to measure the distance of fungal burden to bronchioles by excluding surfaces for the alveoli with diameters less than 50 μm, which is a typical diameter of an alveolus in mice (34, 35) (Fig. 4A). We observed that more than 40% of the fungal burden colocalized with bronchioles (first bin of histogram in Fig. 4C) and that over 20% located in close proximity to bronchioles. Only a few (∼20%) A. fumigatus infection foci positioned at sufficient distance (>100 μm) to be considered inside alveoli (Fig. 4C). This proves that around 80% of the A. fumigatus conidia did not reach deep into murine lung alveoli upon inoculation. We further quantified the ratios of AMs (Fig. 4D) and PMNs (Fig. 4F) that located in bronchioles rather than in the alveoli or other parts of the tissue. Remarkably, we found that a small fraction of AMs localized in the bronchioles in healthy, untreated mice (Fig. 4E), an observation that, to our knowledge, had not been reported until now. Even more interestingly, we found that the ratio of AMs within bronchioles increased upon infection (Fig. 4D), which suggests that AMs from the alveoli were actively attracted toward the foci of infection in bronchioles. Under steady-state conditions, in contrast, there were virtually no PMNs in the bronchioles in C-treated mice and those that were present showed no such attraction (Fig. 4F). We documented an increase of PMNs in the bronchioles in CC and Irr mice; however, these results should not be overinterpreted, as there were extremely low numbers of PMNs present in the lungs of these mice. Once again, we repeated the measurements in an independent experiment, which corroborated the accuracy and reproducibility of LSFM and validated the results (Fig. S4).

10.1128/mBio.02752-19.4 FIG S4 Independent experiment confirming conidia deposition within lungs. To evaluate whether quantitative analysis of LSFM images provides accurate and reproducible data on the localization of conidia (A) and AMs (B) after intranasal inoculation within the airways, we conducted an independent experiment with additional experimental replicates (*n* = 3 mice). (A) Distance distributions of fungi and bronchioles are presented as a histogram for the different treatments. Spores (∼60%) are closely positioned in the vicinity of bronchioles, indicating that a vast majority of the infective conidia did not reach the alveoli. (B) Colocalization of AMs with bronchioles is displayed as a ratio of total cell numbers (*n* = 3 mice and 2 to 3 3D stacks per mouse). One-way ANOVA was applied with Tukey’s multiple comparisons. Download FIG S4, TIF file, 0.2 MB. Copyright © 2020 Amich et al. 2020 Amich et al. This content is distributed under the terms of the Creative Commons Attribution 4.0 International license.

In summary, the precisely detailed architectural information provided by LSFM has provided the first direct evidence that the majority of inhaled conidia do not reach the lung alveoli but instead are deposited in bronchioles. Furthermore, it disclosed a novel location and activity of AMs outside the alveoli. Hence, LSFM has the potential to open new avenues of investigation into the host-pathogen interplay.

## DISCUSSION

In this study, we employed LSFM for the first time to simultaneously investigate the anatomical distribution of the pathogenic fungus A. fumigatus and define the immune cell populations in murine models of pulmonary fungal infection. LSFM proved superior to current gold standard techniques in providing quantitative data in the context of spatial anatomical information from analyses of large volumes of mouse lung tissue. Previously, the fungal burden (i.e., the degree of fungal growth) had been measured by CFU or by qPCR. Along these lines, quantification of the number of immune cells in whole lungs and phenotypic characterization of those cells were performed with flow cytometry. However, those methods require homogenization of the lungs and thus result in destruction of the anatomical context of lung tissues. In contrast, LSFM enabled us to quantify fungal burden in the intact tissue environment and to visualize differentiated A. fumigatus phenotypes and structures of hyphal growth invading the tissue, thus providing a sensitive measure of the stage of disease development in the context of the tissue architecture. Quantitative microscopy of intact lung lobes provided objective parameters to sensitively measure fungal burden (e.g., expressed as volume [percent] of fungus tissue infiltrates) and differentiate growth stage (e.g., hyphal length). As LSFM can visualize and measure even discrete fungal burdens within tissues, this method holds great potential to reshape *in vivo* models to enable the determination of more physiologically appropriate infection doses (i.e., to reduce such doses by several orders of magnitude). Beside the scientific merit, this would also contribute to the increased refinement of animal experimentation by reducing the disease burden according to the 3R principles (replacement, refinement, and reduction for the ethical use of experimental animals). Beyond this, we showed that LSFM can be used concomitantly to quantify the number of defined immune cell populations and to map their locations. 3D fluorescence microscopy of intact lung lobules provides a clear benefit as it minimizes bias and misinterpretations. Analysis does not suffer from sampling and cell extraction biases and, importantly, provides anatomical data that allow calculation of the distribution of the cells in the tissue, their clustering with A. fumigatus, and their phagocytosis rate upon infection. Until recently, the only alternative to obtain lung anatomical information that of performing histology of thin consecutive lung sections, staining them with certain dyes (for instance, periodic acid-Schiff stain [PAS] or Gomori-Grocott stain for fungal hyphae or specific antibodies [to detect, for example, specific cell populations {34}]). Analyses of consecutive histological specimens are time- and labor-intensive; therefore, the volume of tissue that can be investigated within a reasonable time frame is low. Thus, histology sections serve as a reference for fungal germination/hyphal enlargement or presence/absence of immune cells but provide qualitative rather than quantitative data. Recently, Shevchenko and colleagues utilized confocal microscopy to investigate neutrophil recruitment and location in the conducting airway upon A. fumigatus infection (35). In a further development, Kowalski and colleagues utilized the passive clarity technique (PACT) to investigate the architecture of *in vivo* fungal growth in cleared lungs using confocal microscopy (36). Although these studies represent a significant improvement compared with previous techniques, confocal microscopy is still limited by the small fraction of tissue imaged, which makes any quantification and anatomical information of limited value. LSFM permits researchers to image whole lung lobes of infected mice and to capture and quantify the entire host-pathogen interaction. Nevertheless, LSFM has some limitations as well. For example, sample preparation may be difficult when mice need to be perfused; time-consuming, as staining and clearing procedures take several days; and expensive, with large amounts of antibodies being required. Furthermore, the JF5 antibody can detect only actively growing fungus (not resting conidia), which is an advantage for diagnostic purposes but precludes the investigation of very early stages of infection in murine models. Consequently, while this technique has enormous potential, it is still in its infancy with respect to high-throughput or exploratory analyses. In addition, the advantage of LSFM in whole-organ imaging is offset by lower spatial resolution than that of confocal microscopy (15, 37, 38), which enables more-refined subcellular analyses (39). Furthermore, LSFM of the opaque lung tissue also requires tissue fixation and thus allows only snapshots of the infection process. In contrast, methods employing multiphoton microscopy enable live imaging of explanted organs (38, 40–42) or even *in vivo* microscopy (43, 44) using a thoracic window (45) with even if a smaller field of view. Fortunately, as recently demonstrated (14), LSFM and two-photon microscopy can complement each other with high added value such that both tissue architecture and cell dynamics can be interrogated to investigate the dynamic temporal host-pathogen interactions as well as large-scale distributions.

In this study, LSFM proved especially advantageous with respect to visualizing and quantifying fungal growth and the immune response in the lungs of mice treated with various immunosuppressive regimens commonly used to model invasive pulmonary aspergillosis. It has previously been reported that A. fumigatus grows extensively and invades the lung tissue in mice treated with cyclophosphamide and cortisone (CC), whereas its growth is restricted in cortisone (C)-treated mice (21, 22, 46, 47). However, this had previously been inferred only from classical histology sections, as the actual 3D structure of fungal foci in the whole lungs had not been visualized. Here, we show that in corticosteroid-treated mice, A. fumigatus formed condensed foci of growth without hyphal extensions throughout the tissue and the percentage of invaded lung tissue remained very low. In CC-treated mice, in contrast, the fungus created a complex 3D hyphal structure that extended throughout the surrounding tissue, invading a high percentage of tissue volume (average, 1.5% of the whole lung). Interestingly, although fungal hyphae formed in irradiated mice, these comprised only an intermediate fungal architecture that invaded lung tissue at a lower level than was observed in CC-treated mice. Such fungal growth patterns matched the observed effects of the immunosuppressive drugs on AMs and PMNs (48, 49). Neither cell population was depleted in mice treated with corticosteroids; as a result, fungal growth was constrained. However, the function of the AMs appeared to be compromised, as revealed by determination of the numbers of AMs directly colocalizing with A. fumigatus, and infection did not further increase the already high PMN numbers. Therefore, the sustained attempt to eliminate persisting fungus likely contributed to the hyperinflammation characteristics of this model (46, 50, 51). In the CC model, AM numbers were similar to those seen with mice receiving only corticosteroids; this demonstrates that cyclophosphamide treatment did not deplete AMs. Upon infection, AMs also clustered with A. fumigatus, suggesting that CC treatment did not even affect the recognition of A. fumigatus and its interaction of AMs. Notably, the ratio of AMs interacting with A. fumigatus conidia was higher in CC-treated mice than in C-treated mice. It is tempting to speculate that this might be explainable by the lack of a coordinated PMN defense, leaving AMs as the only cell type to interact with conidia. Therefore, it seems that AMs as first responders attempt to contain the infection. If AMs cannot eliminate A. fumigatus, the host relies on an efficient secondary response by PMNs to control the fungus. If PMNs are completely abrogated, as observed in the scenario of CC-treated mice, massive fungal growth culminates in invasive aspergillosis. A. fumigatus grew more extensively in mice receiving an ablative bone marrow dose of irradiation than in the corticosteroid model, likely because there were not sufficient PMN numbers to act as second line of defense; however, the level of growth was slightly lower than in the CC model, which could be explained by the fact that the AMs and the few PMNs present seemed to remain mostly functional to eliminate many conidia (52). Accordingly, irradiated mice displayed fewer foci of fungal growth than CC-treated mice and the more extensively dispersed mycelia indicated that the remaining host immune cells contained the growing hyphae to a certain degree.

LSFM revealed that about 80% of the intranasally administered A. fumigatus spores did not reach deep into the lung alveoli, a result that agrees with the current line of thought concerning the localization of fungal invasion in clinical IPA. Moreover, this finding has important implications for the conclusions that can be reached from murine experiments. For example, elastase has been disregarded as a virulence factor in A. fumigatus (53), but that could be because degrading elastin might be more relevant in the alveoli, where elastin is readily present in the alveolar wall. Besides, the first contact with the host is not orchestrated exclusively by alveolar epithelial cells, such as is normally assumed in humans (54). It is nevertheless important that there are morphological differences between the human and the murine respiratory tracts, with particularly enormous differences in the spatial dimensions, e.g., the distance from the nasal/oral cavities to the tracheobronchial tree or to the surface of an alveolus, which is approximately 20-fold smaller in a murine alveolus (55) than in a human alveolus (estimated mean alveolar volume measures of only <5.95 × 10^4^ μm^3^ in mice [56] compared to 4.2 × 10^6^ μm^3^ in humans [57]). Therefore, the exact localizations of conidia might be slightly different in humans and mice due to anatomical size constrains.

Interestingly, we observed that a small ratio of CD11c^+^ SiglecF^+^ AMs localized outside the alveoli, in the bronchiolar space, a finding that, to the best of our knowledge, had not been reported before. Upon infection, the ratio of AMs in bronchioles increases, suggesting active recruitment toward foci of fungal infection outside the alveolar space. We believe that this is an important result that may open new research avenues and raise interesting questions. Do these cells comprise a distinct AM subset? How are the cells recruited to those sites? What are the distances that they can overcome? Which signals attract them?

In summary, we have shown that state-of-the-art LSFM is a powerful tool that can be used to describe important features of fungal growth and of the local immune response in commonly used murine models of pulmonary aspergillosis. We have demonstrated that LSFM can comprehensively localize all individual foci of fungal growth and that its use permits investigation of local host-pathogen interactions at cellular resolution. This opens the possibility of assaying models of pulmonary aspergillosis using lower, more physiologically appropriate doses of infective conidia while still gaining insight into the immune response. Hence, this report not only advances our understanding of the infection process under conditions of immunosuppression but also could form the basis for refining and maximizing the utility of animal models of pulmonary infection.

## MATERIALS AND METHODS

### Mice.

All experiments were conducted with female 8-to-12-week-old BALB/c mice (Charles River, Sulzfeld, Germany). Mice were maintained in individually ventilated cages (IVC) with *ad libitum* access to water and food. All experiments were performed according to the German regulations for animal experimentation and were approved by the Regierung von Unterfranken (55.2-2531.01-86-13 and 55.2–2532-2-403) as responsible authority.

### Immunosuppression regimens and A. fumigatus infection.

Mice were injected subcutaneously with hydrocortisone acetate (Sigma-Aldrich) (112 mg/kg of body weight) alone (C group) or together with cyclophosphamide (Sigma-Aldrich) (150 mg/kg) intraperitoneally on days –3 and –1 prior to infection (CC group). Mice were myeloablatively irradiated (8 Gy) by the use of an electron linear accelerator (Mevatron Primus; Siemens, Germany) 3 h before infection (Irr group).

On day 0, mice were intranasally infected with 2 × 10^5^ freshly harvested conidia (suspended in 0.9% NaCl plus 0.005% Tween 20) of the A. fumigatus clinical wild-type isolate ATCC 46645. Infection was left to progress for 16 h or 3 days, and mice were then processed for lung harvesting.

### Lung preparation for LSFM.

Prior to lung extraction, mice were perfused using an Ismatec Reglo analog pump (IDEX Health & Science LLC, Oak Harbor, WA, USA) for 2 min with phosphate-buffered saline (PBS) and 8 min with 4% paraformaldehyde (PFA). Lungs were further fixed 2 h in 4% PFA and washed with PBS three times for 30 min each time before processing. To visualize Aspergillus fumigatus, lungs were blocked overnight in PBS–2% fetal calf serum (FCS)–0.1% Triton X and monoclonal antibody JF5 (DyLight 655) against Aspergillus fumigatus was added in a 1:100 dilution. To stain immune cell populations in mouse lungs, the following antibodies were used: anti-CD11b (M1/70) (AF488), anti-CD11c (N418) (AF488), anti-Ly6G (1A8) (DyLight 755), and anti-SiglecF (E50-2440) (DyLight 755) (BioLegend, Uithoorn, The Netherlands, and eBioscience, Frankfurt, Germany). All antibodies were added to the samples at a 1:100 dilution in PBS, and the samples were incubated 2 days at 4°C with gentle shaking. After staining, lungs were washed with PBS three times for 30 min each time, samples were dehydrated in a graded ethanol series (30%, 50%, 70%, 80%, and 90% for 1.5 h each at room temperature and 100% overnight at 4°C). The next day, the samples were rinsed for 2 h in 100% *n*-hexane; *n*-hexane was then replaced stepwise by a clearing solution consisting of 1 part benzyl alcohol in 2 parts benzyl benzoate (Sigma-Aldrich). Air exposure was strictly avoided at this step. Tissue specimens became optically transparent and suitable for the LSFM imaging after incubation in the clearing solution for at least 2 h at room temperature.

### LSFM setup and data acquisition.

The LSFM setup is home-built. For excitation, a customized fiber-coupled laser combiner (BFI OPTiLAS GmbH, Groebenzell, Germany) was used that provided the required excitation lines of 491, 532, 642, and 730 nm. For laser beam collimation (beam diameter = 3 mm), a Hund objective (A10/0.25; Hund, Wetzlar, Germany) was used. A DCLP 660 dichroic beam splitter (AHF Analysentechnik, Tübingen, Germany) combined the two beam paths. A single-axis galvanometer scanner (6210H; Cambridge Technologies, Bedford, MA, USA) in combination with a theta lens (VISIR f. TCS-MR II; Leica, Mannheim, Germany) finally created a virtual light sheet that was additionally pivot scanned by a two-axis resonant scanner system (EOP-SC, 20-20X20-30-120; Laser2000, Wessling, Germany) to minimize shadowing artifacts (58). The light sheet was projected onto the sample via a tube lens and a lens objective (EC Plan-Neofluar 5×/0.16 M27; Zeiss, Göttingen, Germany). The objective on the detection side (HCX apochromatic [APO] L20×/0.95 IMM; Leica, Mannheim, Germany) collected the fluorescence perpendicularly to the light sheet, and, in combination with an infinity-corrected 1.3× tube lens (model 098.9001.000; Leica, Mannheim, Germany) (Infinite/240 to 340), projected the image into a scientific complementary metal oxide semiconductor (sCMOS) camera (Neo 5.5; Andor, Belfast, United Kingdom) (2,560 by 2,160 pixels, 16.6-mm-by-14.0-mm sensor size, 6.5-μm pixel size). The fluorescence was spectrally filtered by typical emission filters (AHF Analysentechnik, Tübingen, Germany) according to the use of the following fluorophores: BrightLine HC 525/50 (Autofluorescence), BrightLine HC 580/60 (Alexa Fluor 532), HQ697/58 (Alexa Fluor 647), BrightLine HC 785/62 (Alexa Fluor 750). Filters were part of a motorized filter wheel [MAC 6000 Filter Wheel Emission TV 60 C 1.0× with MAC 6000 controller; Zeiss, Göttingen, Germany] placed in the collimated light path between detection objective and tube lens. Multicolor stacks were acquired in increments of 2 μm by imaging each plane in all color channels sequentially. Hardware components for image acquisition (laser, camera, lenses) were controlled by IQ 2.9 software (Andor, Belfast United Kingdom). Images were saved as .tiff files and analyzed as described below.

### Analysis of LSFM images.

IMARIS software v8.1.1 (Bitplane AG, CA, USA) was employed to analyze the images obtained with LSFM. When required, background subtraction was applied in accordance with the diameter of the cell population to eliminate unspecific background signals. Fungal and cellular isosurfaces were segmented using the option “Surface” with a smoothing value of 10% of the expected diameter of the object and manual thresholding of the signal. Filaments were calculated from a masked channel from the A. fumigatus isosurfaces, using starting points of 50 to 70 μm and seed points 7 of 3 μm (depending of the degree of fungal invasion) and manual thresholding of the signal. To measure the distance of the fungal burden from bronchioles, we created isosurfaces from lung epithelial cells (podoplanin) signal and filtered surfaces with a diameter less than 50 μm to exclude alveolus structures.

### Cell distribution analysis.

The distribution of stained cells in the light sheet microscopy data resembles a spatial point pattern in mouse lungs (59). To identify the clustering of the cells and characterize the cell distribution patterns, a pair correlation function [*g*(*r*)] (60, 61) was calculated at distance *r* as follows: *g*(*r*) = average number of cells within rings at distance *r* from an arbitrary cell. The pair correlation function can correctly identify the aggregation length scale and distance between clusters of cells in the lung. In cases of random cell distributions, the *g*(*r*) value is about 1. Cells expulsion can be revealed by *g*(*r*) <1, and aggregation is realized by *g*(*r*) >1. A paired signed rank test between infected and uninfected pair correction functions at distances where the strong clustering spatially on *r* = 0 to 100 μm was applied to detect statistically significant aggregation.

### Group size.

We used a previously published method to calculate the group sizes for *in vivo* experiments (62). This method is based on a simple formula derived from the *t* test: *n* = 1 + 2*C*(*s*/*d*)^2^ (where *C* is dependent on values chosen for significance level and power, *s* is the standard deviation, and *d* is the difference to be detected. On the basis of published literature (21–31) and our own results, we determined that the appropriate sample sizes are *n* = 3 to 5 mice for reliable detection of reductions (with a power of 90% and a significance level of 5%) in fungal burden and *n* = 6 to 9 mice for determinations of cell recruitment to the lungs. We have successfully used those group sizes in previous research.

As there was no previously published literature reporting the use of LSFM to investigate the burden of infection and the immune responses, the values for *d* and *s* could not be computed. Therefore, for this study we made the following assumption: considering that LSFM captures the information from whole lungs and does not require processing of the samples, the measurements should be very accurate and the levels of variability low. Hence, the sample size required to obtain statistical power should be smaller than that required for other methods. Consequently, we decided to use two mice per group. To validate this assumption, we repeated selected experiments using an additional 3 mice per group and obtained comparable results (see the figures in the supplemental material).

### DNA extraction from lungs and qPCR analysis.

Explanted, frozen lungs were lyophilized for 48 h in a CoolSafe ScanVac freeze drier connected to a Vacuubrand pump and subsequently ground in the presence of liquid nitrogen. DNA was isolated from the powder using a DNeasy blood and tissue kit (Qiagen). DNA concentrations and quality were measured using a NanoDrop 2000 instrument (Thermo Fisher Scientific). To detect the fungal burden, 350 ng of DNA extracted from each infected lung was subjected to qPCR using SYBR green JumpStar *Taq* ready mix (Sigma-Aldrich). The primers used to amplify the A. fumigatus β‐tubulin gene (AFUA_7G00250) were (forward) 5′-ACTTCCGCAATGGACGTTAC-3′ and (reverse) 5′-GGATGTTGTTGGGAATCCAC-3′. Those designed to amplify the murine actin locus (GenBank accession no. NM_007393) were (forward) 5′-CGAGCACAGCTTCTTTGCAG-3′ and (reverse) 5′-CCCATGGTGTCCGTTCTGA-3′. Standard curves were calculated using different concentrations of fungal and murine genomic DNA (gDNA; pure template). Negative controls containing no template DNA were subjected to the same procedure to exclude or detect any possible contamination. Three technical replicates were prepared for each lung sample. qPCRs were performed using a 7500 Fast real-time PCR system (Thermo Fisher Scientific) with the following thermal cycling parameters: 94°C for 2 min and 40 cycles of 94°C for 15 s and 58°C for 1 min. Data were analyzed using the 7500 software (Thermo Fisher Scientific). The fungal burden was calculated by normalizing the number of fungal genome equivalents (i.e., the number of copies of the tubulin gene) to the murine genome equivalents in the sample (i.e., the number of copies of the actin gene) (63).

### Flow cytometry.

Cell suspensions from murine lungs were prepared as follows. GentleMACS and a lung dissociation kit from Miltenyi Biotec Inc. (130-095-927) were used according to the manufacturer’s protocol to prepare single-cell suspensions for flow cytometry. Briefly, lungs were dissected into single lobes and all the lobes of a mouse were transferred into one gentleMACS C tube containing the mixture of enzymes A and D provided by the manufacturer. Program m_lung_01 was selected, and after termination of this step, C tubes were detached from the genteleMACS dissociator and incubated for 30 min at 37°C on the shaker. Program m_lung_02 was run from the gentelMACS dissociator, and the C tubes were centrifuged shortly after the termination of this program. The dissociated tissues were filtered through a cell strainer (Greiner Bio-One) (70-μm pore size). Suspensions were further treated with fluorescence-activated cell sorter (FACS) lysis buffer (8.99 g/liter NH_4_Cl, 1 g/liter KHCO_3_, 0.037 g/liter EDTA) for 2 min. Cell suspensions were centrifuged and resuspended in 1 ml of PBS. Single-cell suspensions were stained with a LIVE/DEAD Fixable Violet dead cell stain kit (Molecular Probes, Life Technologies) to exclude dead cells and with antibodies anti-SiglecF (clone E50-2440, conjugated to Alexa Fluor 647) and anti-CD11c (clone N418, conjugated to phycoerythrin [PE]-Cy7). Flow cytometry was performed on a Attune NxT Flow Cytometer (Thermo Fisher Scientific), and data were analyzed with FlowJo Software version 10 (Tree Star, Ashland, OR). Gates were set using the Fluorescence Minus One gating strategy (64). Antimouse CompBeads (BD) were used for compensation controls.

### Statistical analysis.

Ordinary one-way analysis of variance (ANOVA) was used with Tukey’s multiple comparisons for normally distributed data. Nonnormal data were compared employing the Kruskal-Wallis test. All statistical analysis was performed with GraphPad Prism Version 7.
