# Supplementary figures and images for: Three-Dimensional Light Sheet Fluorescence Microscopy of Lungs To Dissect Local Host Immune-Aspergillus fumigatus Interactions
Source: mBio. 2020 Feb 4;11(1):e02752-19. doi: 10.1128/mBio.02752-19 (PMC7002341; doi:10.1128/mBio.02752-19)

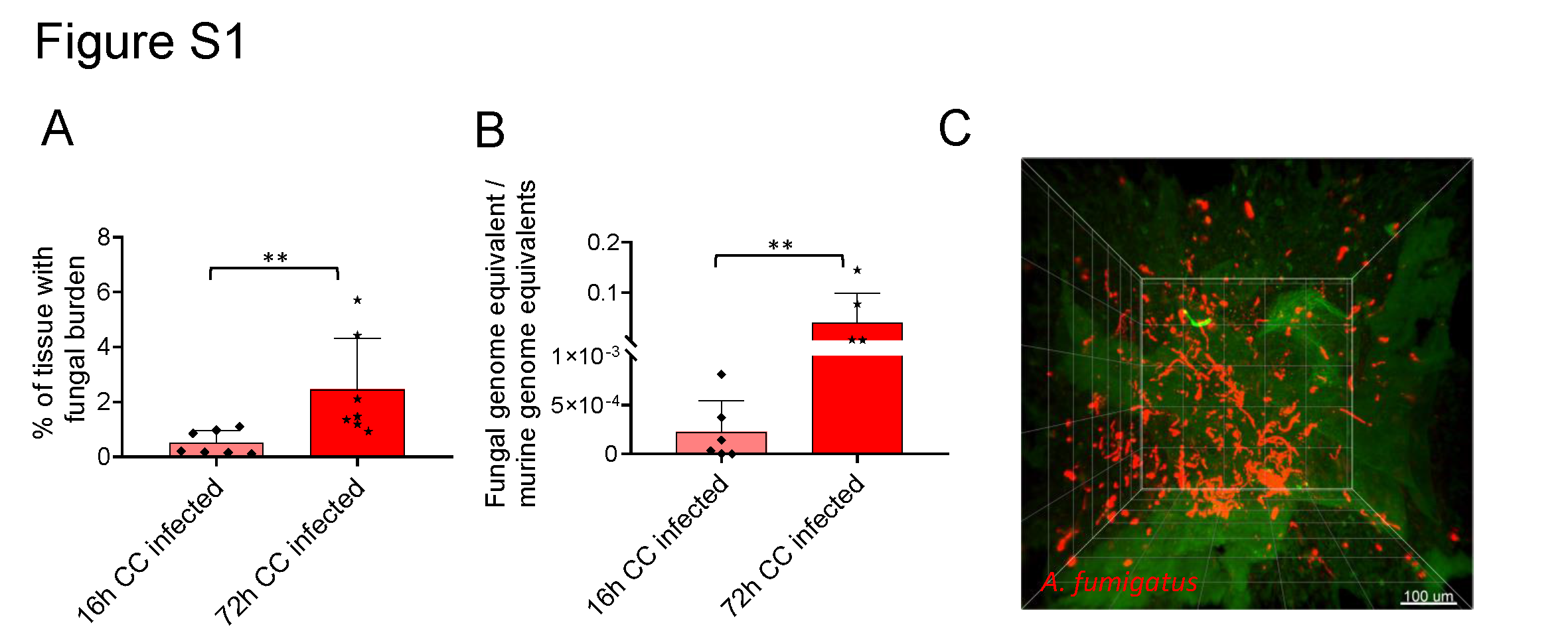

Supplement: FIG S1 [file mBio.02752-19-sf001.tif]

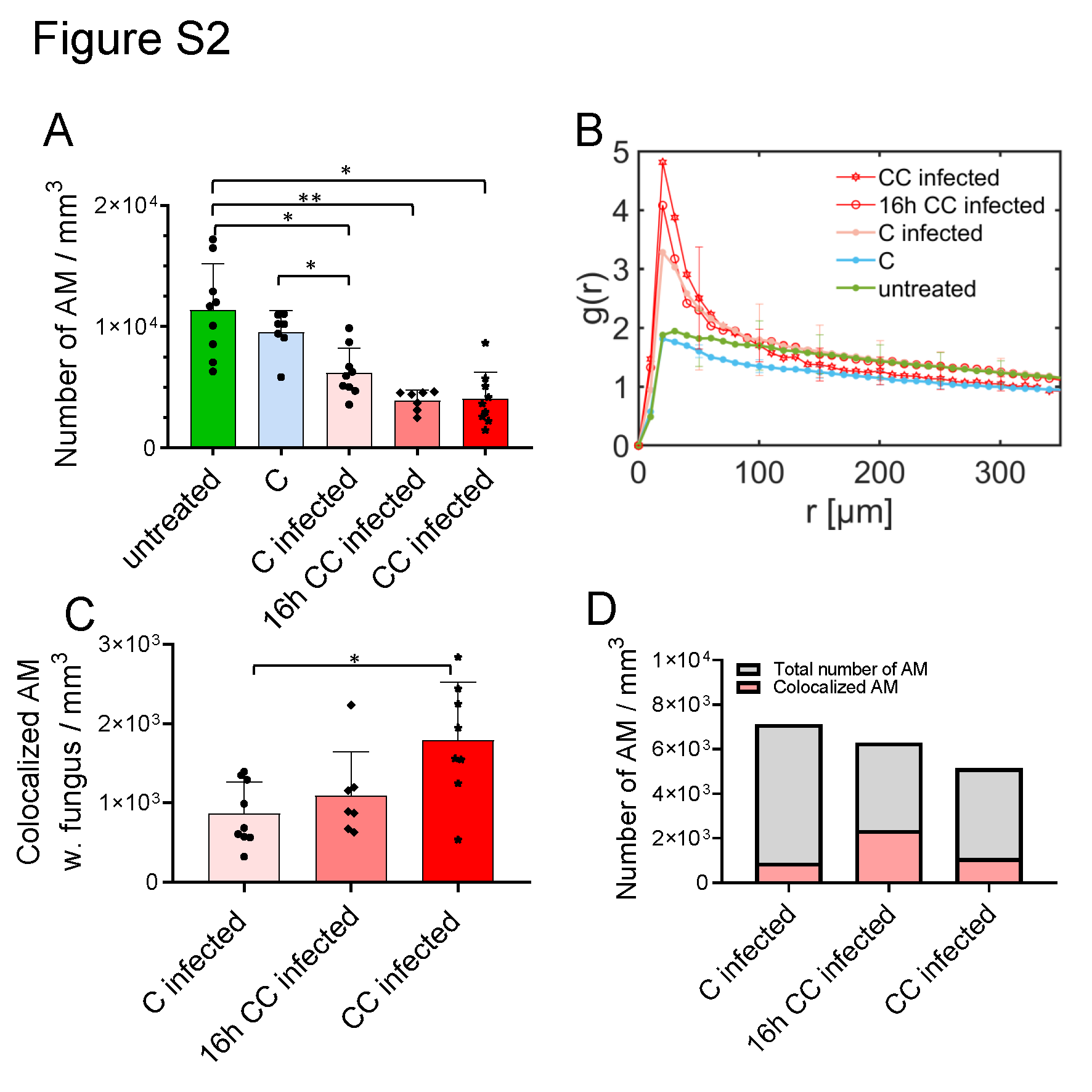

Supplement: FIG S2 [file mBio.02752-19-sf002.tif]

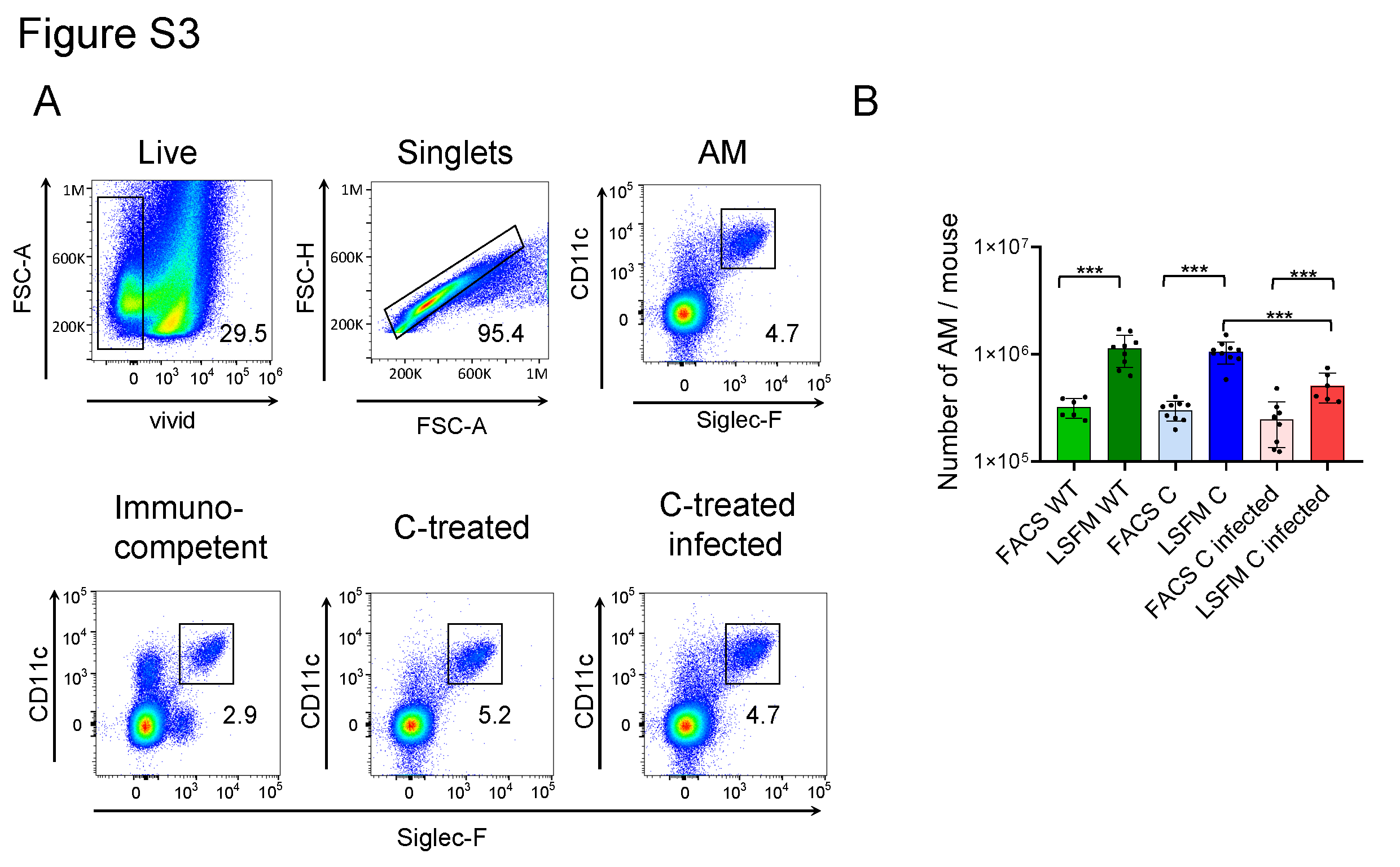

Supplement: FIG S3 [file mBio.02752-19-sf003.tif]

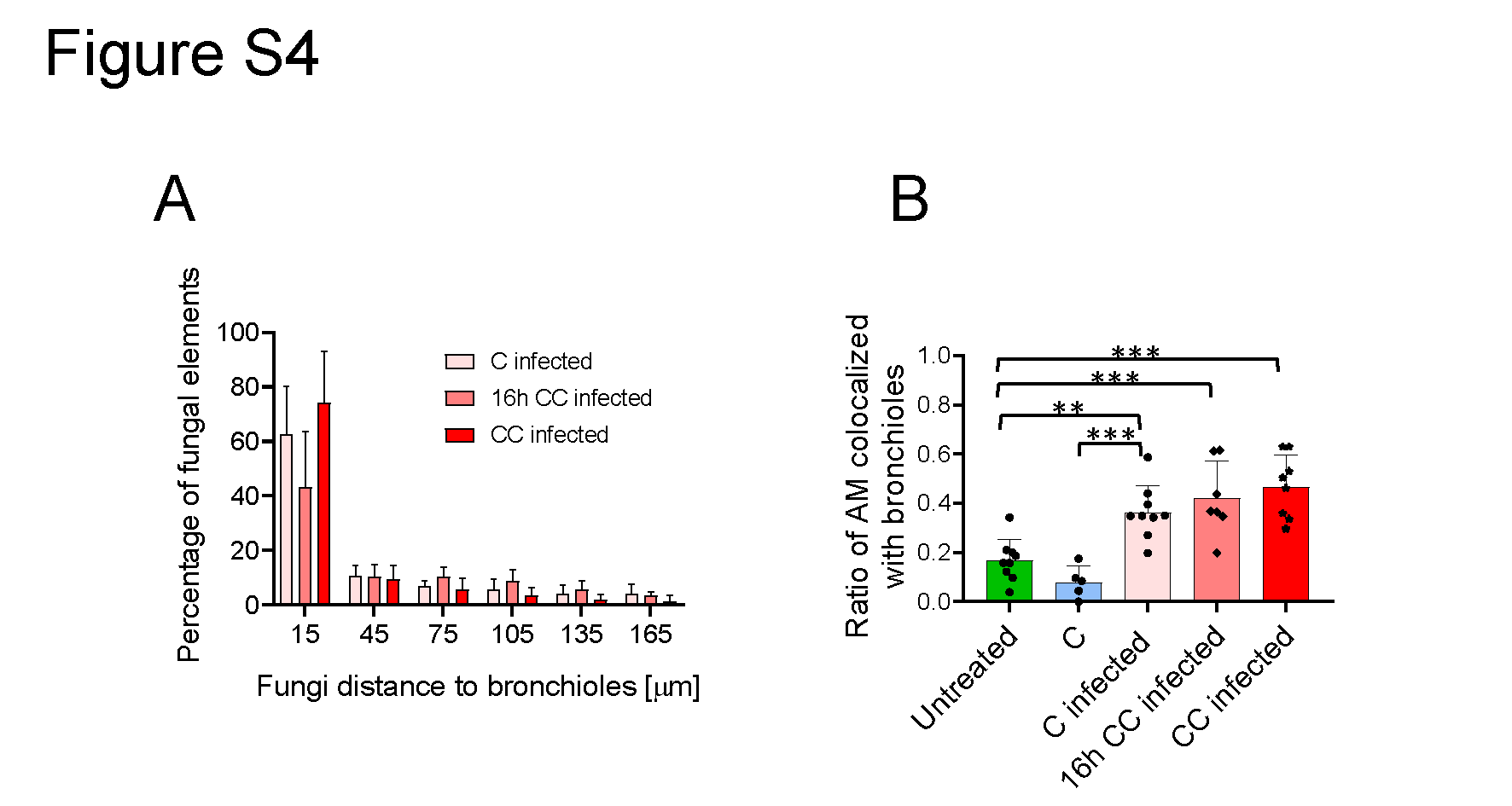

Supplement: FIG S4 [file mBio.02752-19-sf004.tif]
